# Nano-MnO_2_ Decoration of TiO_2_ Microparticles to Promote Gaseous Ethanol Visible Photoremoval

**DOI:** 10.3390/nano8090686

**Published:** 2018-09-03

**Authors:** Marta Stucchi, Daria C. Boffito, Eleonora Pargoletti, Giuseppina Cerrato, Claudia L. Bianchi, Giuseppe Cappelletti

**Affiliations:** 1 Chemistry Department, University of Milan, via Golgi 19, 20133 Milano, Italy; eleonora.pargoletti@unimi.it (E.P.); claudia.bianchi@unimi.it (C.L.B.); giuseppe.cappelletti@unimi.it (G.C.); 2 Chemical Engineering Department, Ècole Polytechnique Montréal, Pavillon Principal, Centre-Ville, Montréal, QC H3C 3A4, Canada; daria-camilla.boffito@polymtl.ca; 3 National Consortium for Materials Science and Technology (INSTM), via Giusti 9, 50121 Florence, Italy; 4 Chemistry Department & NIS Centre, University of Turin, via Pietro Giuria 7, 10125 Turin, Italy; giuseppina.cerrato@unito.it

**Keywords:** micrometric TiO_2_, Mn decoration, visible light photocatalysis, impregnation pH, surface hydroxyl groups

## Abstract

TiO_2_-based photocatalysis under visible light is an attractive way to abate air pollutants. Moreover, developing photocatalytic materials on a large-scale requires safe and low-cost precursors. Both high-performance TiO_2_ nanopowders and visible-light active noble metals do not match these requirements. Here, we report the design of novel Mn-decorated micrometric TiO_2_ particles. Pigmentary TiO_2_ replaced unsafe nano-TiO_2_ and firmly supported MnO_x_ particles. Mn replaced noble metals such as Au or Ag, opening the way for the development of lower cost catalysts. Varying Mn loading or pH during the impregnation affected the final activity, thus giving important information to optimize the synthesis. Photocatalytic activity screening occurred on the gas-phase degradation of ethanol as a reference molecule, both under ultraviolet (UV) (6 h) and Light Emitting Diode (LED) (24 h) irradiation. Mn-doped TiO_2_ reached a maximum ethanol degradation of 35% under visible light after 24 h for the sample containing 20% of Mn. Also, we found that an acidic pH increased both ethanol degradation and mineralization to CO_2_, while an alkaline pH drastically slowed down the reaction. A strict correlation between photocatalytic results and physico-chemical characterizations of the synthesized powders were drawn.

## 1. Introduction

Environmental photocatalysis based on TiO_2_ and its applications for pollution abatement relies on some key aspects: (i) The availability of inexpensive and stable TiO_2_ [1]; (ii) the photoelectric effect, for which photocatalysis depends upon the energy of the incident photons, but not on their intensity (thus, even a small amount of photons of the required energy induces photocatalysis) [2]; (iii) the effectiveness of photocatalysis in abating pollution as it accumulates, as opposed to treating large volumes [3,4].

In 2001, Wolkoff and Nielsen [5] estimated indoor concentrations for twelve Volatile Organic Compounds (VOCs). Concentrations of organics such as benzene, ethylbenzene, and *o*-xylene were less than 5 µg m^−3^; toluene and xylene isomers, instead, exceeded 5 µg m^−3^, with concentrations greater than 15 µg m^−3^ in most cases. Recently, Höllbacher et al. [6] studied indoor air quality considering specific human activities, and they monitored VOCs, particulate matter (PM), carbonyl compounds and CO_2_. The highest VOC concentration recorded was around 3500 µg m^−3^; they detected a maximum formaldehyde concentration of 76 µg m^−3^, and the PM concentration was 378 µg m^−3^. The concentration level of many pollutants changes the incidence of diseases among people who spend time inside buildings [7].

The conventional air-purification systems are usually provided with filters. However, in such filters, pollutants can be accumulated. Moreover, treatment of old filters could cause the risk of secondary pollution. Although TiO_2_ is excellent to photocatalytically cleave organic compounds, photocatalysis appears as hardly applicable indoors, because visible light is not sufficient to activate TiO_2_. Indeed, anatase TiO_2_ absorbs ultraviolet light with wavelengths shorter than 380 nm [8]. Considering the sunlight, the Ultraviolet (UV) content barely covers 5% of the total irradiation spectrum, whereas the ultraviolet light in indoor illumination is significantly smaller because the fluorescent lamps mainly emit visible light [9]. Moreover, considering the growing concerns about energy consumption utilization, Light Emitting Diode lamps (LEDs) are increasingly replacing all other kinds of classical lighting system. LED emits in the range of 400–700 nm wavelengths only, without any UV component (IUPAC Gold Book, Light-emitting diode, LED). The number of papers regarding the modification of TiO_2_ to improve its photocatalytic properties is countless. Noble metal nanoparticles are actually the only promising way for the enhancement of TiO_2_ properties in the visible spectrum. For example, Pan and Xu [10] deposited noble-metal nanoparticles (Ag, Pt, and Pd) on a TiO_2_ surface. The resulting M-TiO_2_ (M = Ag, Pt, and Pd) nanocomposites were visible-light active for the selective oxidation of benzyl alcohol. Elahifard et al. [11] synthesized Ag/AgBr/TiO_2_ covered apatite and proved their high photocatalytic activity as anti-bactericidal under visible light. Also, Au/TiO_2_ catalysts degraded formaldehyde under visible light in a single pass continuous flow reactor, with conversions above 80% [12].

However, the high cost of metals such as Au, Ag, and Pt, limits the large-scale production of photoactive TiO_2_-based building materials for pollution abatement in indoor environments. The use of some transition metal oxides could increase the photocatalytic activity. Among them, manganese oxides (MnO_x_) recently emerged as quite active in the degradation of organic (VOCs) or inorganic (NO_x_) pollutants, as reported in the recent literature [13]. Since MnO_2_ is rich in non-stoichiometric oxygen vacancies, it could be exploited to increase the carrier density and electrical conductivity of semiconductors [14,15,16]. Mn can form different oxides, such as Mn_3_O_4_, Mn_2_O_3_, and MnO_2_ that show different behavior in relation to the catalytic reaction in which they are involved [17,18,19].

Papers report several synthetic procedures to obtain Mn-TiO_2_ nanoparticles from Ti-based precursors [20,21,22]. Binas et al. [20] reported a sol–gel method for the synthesis of Mn-doped TiO_2_ photocatalysts with a grain size ranging between 20 and 30 nm, active for degradation of Methylene Blue under visible light. Xue et al. [21] synthesized mesoporous structured MnO_2_/TiO_2_ by a modified sol–gel method, showing excellent adsorption properties over the entire visible light range. Another recent paper reported the sol-gel synthesis of 20 wt.% Mn/TiO_2_ nanocomposites at different pH. The nanocomposite synthesized at pH  =  7, which was smaller in crystal size and had a larger pore volume, was the most active in methylene blue degradation. This study showed that pH influenced the amount of dopants on TiO_2_, the amount of OH functional groups on its surfaces, crystal sizes, and pore volume [22].

On the basis of this preliminary research and with the aim of replacing nanopowders with safer micro-TiO_2_, here we report for the first time Mn-decorated samples by impregnation of micro- pigmentary TiO_2_ particles at different pH. We studied the photocatalytic activity both under UV and LED irradiation, selecting ethanol as a model VOC molecule. All the powders were finely characterized and a strict correlation between their physicochemical properties and photoactivity was drawn.

## 2. Materials and Methods

All chemicals were of reagent grade purity and were used without further purification; doubly distilled water passed through a Milli-Q apparatus that was used to prepare solutions and suspensions. Manganese (II) nitrate tetrahydrate (Mn(NO_3_)_2_·4H_2_O, 99%, Sigma-Aldrich, Sigma-Aldrich SRL, Milan, Italy) was the salt precursor; the impregnation procedure required acetone (CH_3_COCH_3_) (≥99.9%, Sigma-Aldrich, Sigma-Aldrich SRL, Milan, Italy), potassium hydroxide (KOH), and hydrochloric acid (HCl) (reagent grade, 37%, Sigma-Aldrich, Sigma-Aldrich SRL, Milan, Italy) to set the acidic or alkaline pH. Micrometric TiO_2_ (by Kronos, identified with the acronym 1077) consists of 100% anatase spherical primary particles of 130 nm, with a surface area of 11 m^2^ g^−1^, as reported by Bianchi et al. [23].

### 2.1. Synthesis of Mn-Impregnated Titania Samples

The Mn-modified samples were obtained through an impregnation method: (i) A fixed amount (1 g) of 1077 was suspended in an aqueous solution (to cover completely the 1077 powder layer at the bottom of the glass flask) with a few drops of acetone in order to increase the wettability of the titania particles; (ii) then, a Mn(NO_3_)_2_·4H_2_O solution was added with a different Mn weight amount (in the range 5–30%). The natural pH of the initial suspension was 4. The dried Mn-doped TiO_2_ particles were obtained by means of a Rotavapor for 26 h (at 40 °C for 24 h and 80 °C for the final 2 h), followed by a thermal treatment in an oven at 100 °C overnight. Every sample was calcined at 400 °C for 2 h in air.

Further, fixing the Mn amount to 20%, the pH of the suspension changed from the spontaneous value (around pH 4) to both acidic (pH 3) and alkaline (pH 9, 12) by addition of HCl and KOH, respectively. The solvent evaporation procedure and the heating treatment were the same as previously reported [24].

The samples were labeled as to Mn_x_1077_y, where x is the Mn weight percentage and y is the pH value.

### 2.2. Physico-Chemical Characterizations

X-ray powder diffraction (XRPD) (Malvern Panalytical Srl, Milan, Italy) analysis was performed on a Philips PW 3710 Bragg–Brentano goniometer equipped with a scintillation counter and 1° divergence slit, 0.2 mm receiving slit, and 0.04° soller slit systems. We employed graphite-monochromated Cu Kα radiation (Cu Kα1 λ = 1.54056 Å, Kα2 λ = 1.54433 Å) at 40 kV × 40 mA nominal X-rays power. Diffraction patterns were collected between 20° and 80° with a step size of 0.1°.

A JEOL 3010-UHR Instrument (acceleration potential 300 kV, LaB6 filament) (JEOL Ltd., Milan, Italy) was utilized to acquire the High Resolution-Transmission Electron Microscopy (HR-TEM) images to evaluate the morphology of all the adopted catalysts.

The BET surface area (S_BET_) (Beckan Coulter, Indianapolis, IN, USA) was determined from nitrogen adsorption–desorption isotherms at 77 K, by using Coulter SA 3100 apparatus.

Diffuse Reflectance spectra (DRS) (Shimadzu Europe, Duisburg, Germany) of the nanopowders were measured on a UV–Vis spectrophotometer Shimadzu UV-2600 equipped with an integrating sphere; BaSO_4_ was used as a “total white” reference.

X-ray Photoelectron Spectroscopy (XPS) (Thermo Scientific, Massachusetts, USA) analysis was carried out by means of an M-probe apparatus (Surface Science Instrument), using a monochromatic Al Kα radiation source (1486.6 eV). The XPS binding energy scale was charge corrected using the standard calibration, fixing the C-1s peak at 284.6 eV.

### 2.3. Photocatalytic Tests

Ethanol (CH_3_CH_2_OH) was selected to evaluate the photocatalytic properties of the Mn-modified TiO_2_ samples in the gaseous phase. The starting pollutant concentration was 400 ppm. The photodegradation reaction was performed in a four openings PIREX glass reactor of 5 L. A gas chromatograph (Agilent 3000 A Micro-GC, Santa Clara, CA, USA), directly connected to the reactor, monitored the internal concentration of the organic molecule, as well as of any by-products and CO_2_, as reported in our previous work [24]. Two different irradiation sources, a UV lamp (HG 500 Jelosil SRL, 315–400 nm and irradiation intensity of 30 W m^−2^, Milan, Italy) and an LED one (MW Mean Well, 400–700 nm, 350 mA, 16.8 W and irradiation intensity of 15,000 lx, to simulate the visible light, Taiwan, China) were used. According to the adopted source, photocatalytic tests lasted 6 h and 24 h, respectively. A hygrometer was placed inside the reactor to continuously monitor the relative humidity (RH around 40% for the whole reaction).

All the Mn-TiO_2_ powders, suspended in 2-propanol, were deposited on a 100 cm^2^ glass slab to obtain a homogeneous thin film. The covered glass support was placed at the bottom of the reactor. A cleaning procedure through the exploitation of an inert gas was performed to remove CO_2_ traces. The desired amount of the organic pollutant was introduced through a microliter syringe.

Dark tests together with photolysis experiments (both under UV and visible light) were carried out to evaluate the molecules adsorption and their direct degradation, respectively. For both the irradiation sources, ethanol adsorption and photolysis were always negligible (<2%).

## 3. Results and Discussion

### 3.1. Ethanol Photodegradation Under UV and LED Sources

Firstly, Mn_x_1077_4 (X refers to the Mn%, while number 4 refers to pH, with x in the range 5–30%) powders were tested on the photodegradation of gaseous ethanol molecules both under UV and visible light. Star Mn_x_1077_4 ting from the ultraviolet-assisted experiments, as already stated in our previous work [23], the abatement of the pollutant and its complete conversion to CO Mn_x_1077_4_2_ within Mn_x_1077_4n 6 h, see Table 1, were reached by bare TiO_2_ (1077), notwithstanding the micrometric distribution of primary particles and aggregates (see Section 3.2). On the contrary, by impregnating the pristine TiO_2_ with Mn, the photocatalytic performance drastically decreased, upon increasing dopant loading, see Table 1, 2nd and 3rd columns. As reported in the literature, the OH species distributed onto the TiO_2_ surface mostly affect the photocatalytic activity [22,25]. Thus, the partial covering of the active sites, which can be activated by UV light, is much more adverse compared to the increment given by Mn in terms of electron capture.

Further, the aim of the present work was the exploitation of the prepared photocatalysts under LED light. As expected, pure 1077 sample showed a scarce amount of activity under visible light (negligible ethanol molecules degradation and mineralization after prolonged irradiation; Table 1, 4th and 5th columns). Instead, by either comparing both the disappearance and mineralization of Mn-doped TiO_2_ or considering the different Mn loadings, Mn_20_1077_4 powder exhibited the best photocatalytic performance (Table 1, 4th and 5th columns). Moreover, EtOH degradation was lower for Mn loadings higher than 20% (Table 1, Mn_30_1077_4 sample). Again, as widely reported, the decoration of the TiO_2_ surface by Mn-based nanoparticles (NPs) increased the visible light absorption but partially covered the active surface sites [22]. Indeed, Mn NPs and active sites have a synergistic effect on the final photocatalytic activity. Thus, when both contributions are balanced, the maximum efficiency is achieved, as reported in the recent literature [26]. Metal and metal oxides species can positively act on the photoactivity of TiO_2_, as demonstrated in the case of Cu by Chiang and Doong [27], because they are able to capture more photons from visible light, leading to the formation of more electron-hole couples, as we also showed in our previous work [26]. Therefore, in a similar way, Mn and MnO_2_ species deposited on the micrometric anatase particles could act as electron traps [28], limiting the e^−^/h^+^ recombination. Moreover, whatever species modifies the bare TiO_2_ support strongly affects the material properties, especially in relation to its amount. Indeed, the dopant content in each TiO_2_-matrix sample is related to the efficiency of the electron-hole recombination process, as well as to the formation of the hydroxyl radicals under an irradiation source [29].

The photocatalytic oxidation pathway was demonstrated to be a two-step process, where acetaldehyde is the main intermediate molecule, and CO_2_ and H_2_O the final products [30,31,32]:

CH_3_CH_2_OH → CH_3_CHO → 2CO_2_

**Figure 1:**
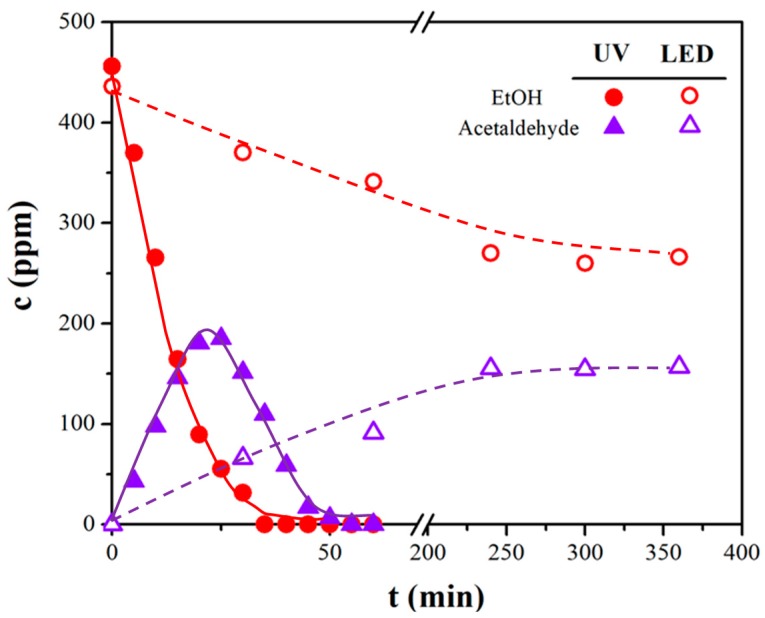
Ethanol disappearance and acetaldehyde formation in the case of Mn_20_1077_4 under both UV and LED sources.

Figure 1 corroborates the previous mechanism showing how the progressive UV-assisted photodegradation of ethanol (in the case of Mn_20_1077_4, as a representative sample) led to the formation of an acetaldehyde by-product and then to the complete conversion to CO_2_. Instead, by means of LED irradiation a drastic decrease of the starting molecule degradation occurred accompanied by a slow acetaldehyde production and partial mineralization, see Table 1, 5th column.

**Figure 2:**
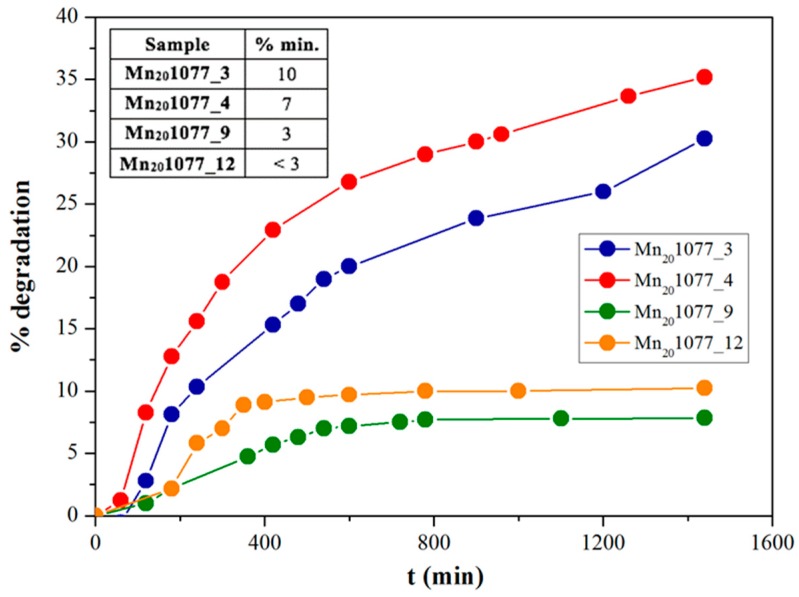
Ethanol degradation percentages after 24 h of Light Emitting Diode(LED) photocatalysis, by different pH Mn-impregnated TiO_2_ samples. Table in inset: Relative mineralization degrees.

The second part of the research work was devoted to evaluating the role of the impregnation pH on the photocatalytic activity. Based on the promising results obtained with manganese-doped powders under LED light, the 20% Mn loading (Mn_20_1077_y series) was selected. A different pH impregnation environment seemed to influence the final ethanol photodegradation, see Figure 2.

Particularly, an acidic pH (samples Mn_20_1077_3 and Mn_20_1077_4) led to an increase of both EtOH disappearance and the degree of mineralization (up to 35% and 10%, respectively for Mn_20_1077_3; Figure 2 and Table 1 in inset). On the contrary, moving towards alkaline values, the overall photocatalytic performances seemed to be drastically inhibited (down to 7% and <3% for disappearance and mineralization by Mn_20_1077_12, respectively). To explain this phenomenon, we believe that the adsorption of ethanol molecules is mostly dependent on the concentration of hydroxyls groups on the surface of the catalyst. Thus, in order to evaluate the role played by either Mn amount or the pH of impregnation on the structural, morphological, and optical properties, several physicochemical analyses were performed.

### 3.2. Mn-TiO_2_ Physico-Chemical Properties

**Figure 3:**
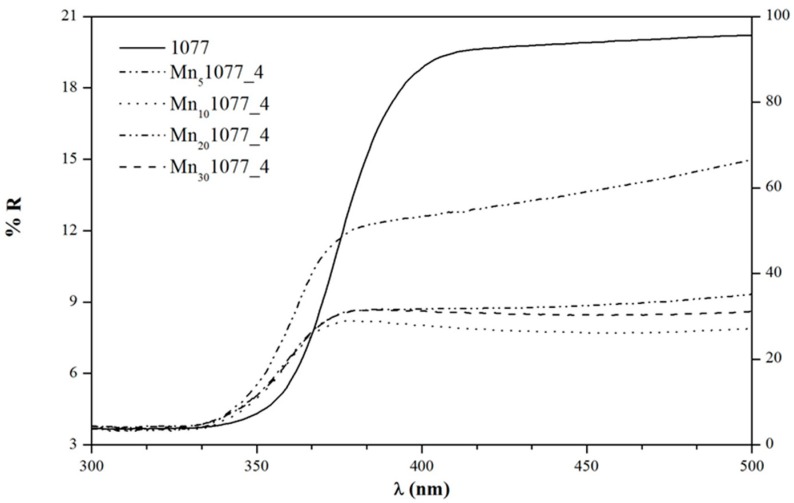
Diffuse reflectance spectra of impregnated TiO_2_ powders with different Mn-dopant concentrations.

As a key parameter to explain the photoactivity of a catalyst, especially in the visible region, band gap evaluation by diffuse reflectance spectra (DRS) analysis was performed and is shown in Figure 3.

As expected, considering the ethanol degradation results under LED light, an increasing amount of manganese led to a decrease of the band gap values, see Table 2, 2nd column and Figure 3. This reduction should improve the photocatalytic performance of doped TiO_2_ particles. On the contrary, varying pH did not seem to influence the optical properties, as shown in Table 2. Hence, the sole DRS measurements were not enough to explain the photocatalytic performances under LED light by varying either the Mn content or the impregnation pH, see Figure 2.

**Figure 4:**
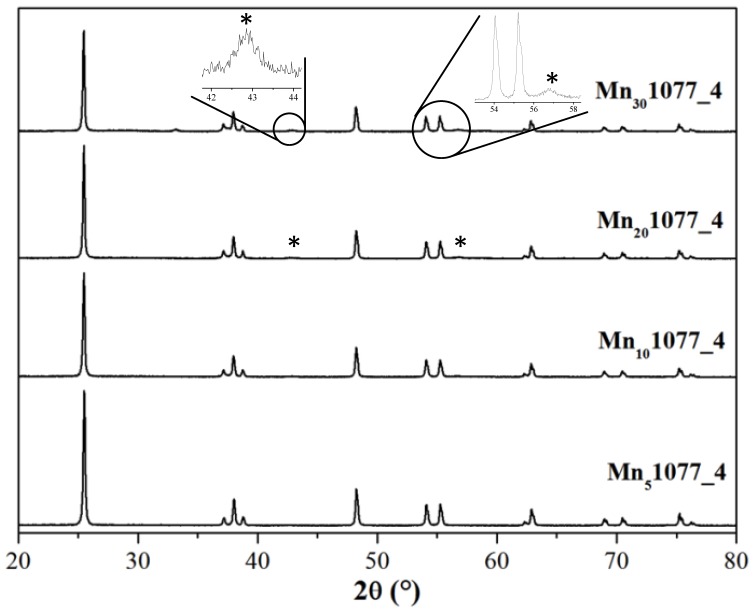
X-ray diffraction lines of Mn_x_1077_4 samples (* = MnO_2_ pyrolusite polymorph).

From a structural point of view, X-ray diffraction patterns, shown in Figure 4, revealed that the Mn content did not affect the TiO_2_ phase composition. Indeed, all patterns show the peak at around 25° ascribable to the anatase phase (ICDD Anatase file No. 21-1272). Interestingly, for Mn amounts higher than 10%, peaks at 2θ around 43° and 56° appeared, as shown in Figure 4, which could be assigned to pyrolusite MnO_2_ species. Instead, pH did not significantly induce structural modifications.

**Figure 5:**
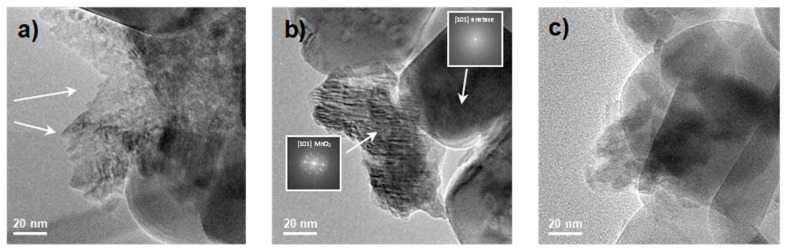
Transmission Electron Microscopy (TEM) images of (**a**) Mn_10_1077_4; (**b**) Mn_20_1077_4; and (**c**) Mn_20_1077_12.

Then, HR-TEM analyses were performed to evaluate the morphological features of the photocatalysts, see Figure 5. All samples showed the peculiar characteristics of the bare TiO_2_ material, which is made up of ordered particles with large average size, namely in the micrometric range (50–100 nm or more in diameter) and smoothed roundish contours: The (101) family of anatase crystal planes (0.352 nm; ICDD anatase file No. 21-1272) [33,34] was observed, see the electron diffraction pattern reported in Figure 5b, which is relevant for all the ordered TiO_2_ microcrystals present in Figure 5. The TiO_2_ morphology remains apparently unmodified for Mn amounts below 10%, see Figure 5a, even though in some regions extra morphological features start to appear, as indicated by the arrows. For higher concentrations (Mn_20_1077_4, Figure 5b), the lattice fringes relative to the (101) family of crystal planes belonging to the pyrolusite MnO_2_ polymorph (0.240 nm; ICDD 24-0735) could be observed (as also confirmed by the electron diffraction pattern reported on the left-hand side of the image), thus corroborating the previous XRPD results. Hence, we could hypothesize that a small presence of this secondary oxide may enhance the photodegradation performances, as previously discussed.

Moreover, in acidic experimental conditions (Mn_20_1077_4 as a representative sample in Figure 5b), TiO_2_ particles did not exhibit any morphological changes, whereas the Mn species were very evident as small envelopes of thin, closely packed particles (average dimensions 3–6 nm) with clear fringe patterns of pyrolusite MnO_2_. In addition, with alkaline impregnation pH, see Figure 5c, a second family plane (111) of manganese dioxide polymorph was also observable.

Furthermore, BET analyses showed relatively low values of surface area for all the adopted samples, confirming the micrometric texture of the aggregates; a slight increase was appreciable for Mn-impregnated TiO_2_ (from 10 to 16 m^2^ g^−1^; Table 2, 3rd column). However, at alkaline pH, this parameter decreased down to 7 m^2^ g^−1^ for the Mn_20_1077_12 sample, see Table 2. These results could be explained by taking into account the actual Mn content obtained by both EDS and XPS analyses, see Table 2, 4th column. The bulk amount of manganese species was consistent with the one expected from the synthetic procedure for all the Mn concentrations. Regarding the impregnated samples at different pH, the interactions between the TiO_2_ micrometric particles and the adopted Mn(NO_2_)_3_ salt precursor were deeply influenced by the pH value. Indeed, the final Mn bulk amount is similar to the starting concentration at an acidic pH, while it decreases at alkaline pH values, see Table 2, 4th column.

On the contrary, the surface Mn/Ti ratios were always higher than those obtained by the EDS analyses in accordance with the experimental method (impregnation) to decorate the micrometric titania powders. Then, notwithstanding the low amount of Mn at alkaline pH, all the guest metal was segregated to the surface forming a dense coverage of MnO_2_ nanopowders. According to Li et al. [35], when manganese content increases, it becomes the recombination center for electron–hole pairs decreasing the separation efficiency of photo-generated charges, therefore invariably reducing/preventing the formation of hydroxyl radicals.

**Figure 6:**
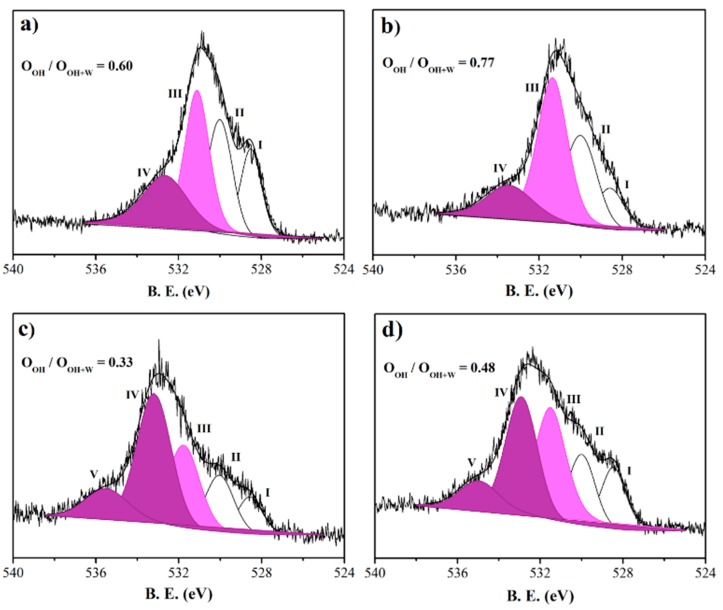
XPS high-resolution spectra of oxygen (O1s) relative to (**a**) Mn_20_1077_3; (**b**) Mn_20_1077_4; (**c**) Mn_20_1077_9; and (**d**) Mn_20_1077_12. Inset: Atomic ratio between the oxygen of surface OH groups (O_OH_, pink area), and the sum of the previous one and the oxygen of the chemisorbed water molecules (O_W_, violet areas).

Hence, to corroborate the previous data and for a complete comprehension of the photocatalytic results, high-resolution O 1s spectra, shown in Figure 6, were fitted using four or five Gaussian/Lorentzian functions superimposed to a Shirley background. On the basis of the recent literature, the peaks could be assigned to; (i) oxygen bound to the metal ions (Ti [36,37] or Mn [38]) in the lattice (peak I at 528.5 eV); (ii) a high binding energy component (HBEC) developed with the increasing loss of oxygen or creation of oxygen vacancies (peak II at 530.0 eV) [39,40]; (iii) a low binding energy component (LBEC) due to adsorption of OH^−^ on the surface (peak III at 531.1 eV) [37,39]; and (iv) water adsorption (peaks IV and V at binding energy (B.E.) higher than 532.0 eV) [37,41]. It is worth noting that the sequence of the ratios between the oxygen of surface OH groups (peak III, pink area), and the sum of the previous one and the oxygen of the chemisorbed water molecules (peak IV and V, violet areas) at increasing pH was fully in accordance with the photocatalytic activities (Mn_20_1077_9 < Mn_20_1077_12 < Mn_20_1077_3 < Mn_20_1077_4, see Figure 2). Hydroxyl groups on the surface of a photocatalyst play an important role in the photocatalytic reaction since they can capture the photo-induced holes from the surface of the material, inhibiting electron–hole recombination as well as forming hydroxyl radicals with high oxidation potentials [42,43]. Thus, at alkaline pH, the high surface Mn loading and the paucity of OH groups inhibit the photocatalytic activity.

## 4. Conclusions

Mn species activates anatase micro-TiO_2_ for the visible light photodegradation of organic pollutants. Particularly, the final photocatalytic performance strongly depends on the Mn amount. Indeed, we surveyed Mn loadings between 5% and 30% under both UV and LED irradiation. Nevertheless, bare 1077 powder was the best performing photocatalyst, and the increasing Mn content led to a drastic decrease of the ethanol photoremoval under UV light. Mn_20_1077_4 showed the highest EtOH degradation under visible wavelengths. In fact, the presence of Mn species provoked a reduction of the band gap value, thus leading to higher efficiency under LED light.

A further phase of the present work was the investigation of the impregnation pH on the final photocatalysts performance. Only a fixed concentration of Mn (i.e., 20%) at an acidic pH (3 and 4) resulted in more active samples, whereas alkaline pH (up to 12) reduced the photocatalytic performance. Indeed, we believe that the powders prepared at a higher pH of impregnation are less active because of the segregation of a high amount of Mn species and concomitantly a low amount of surface hydroxyl groups.

The novel results reported herein are a step forward in the photodegradation of the indoor gaseous pollutants by a micrometric Mn-decorated TiO_2_ catalyst under visible light. Moreover, the positive effect obtained at an acidic pH could be further investigated, to assess whether lower values improve the final catalytic performance.

## Figures and Tables

**Table 1:** Gaseous ethanol photodegradation and mineralization degrees by both pure and Mn-doped 1077 samples, under ultraviolet (UV) (after 6 h) and Light Emitting Diode (LED) (after 24 h) irradiation sources.

Sample	UV (After 6 h)		LED (After 24 h)	
	% Degradation	% Mineralization	% Degradation	% Mineralization
1077	100	100	<2	<2
Mn_5_1077_4	51	14	6	<2
Mn_10_1077_4	50	13	12	<2
Mn_20_1077_4	39	8	35	7
Mn_30_1077_4	10	<2	10	<2

**Table 2:** Band gap values (by diffuse reflectance spectra (DRS)), specific surface area values (S_BET_) and manganese-to-titanium (Mn/Ti) atomic ratios (by EDS and XPS) for all the adopted samples.

Sample	Band Gap (eV)	*S_BET_* (m^2^ g^−1^)	Mn/Ti	
			EDX	XPS
1077	3.28	10	–	–
Mn_5_1077_4	3.05	15	0.04	0.06
Mn_10_1077_4	2.96	14	0.09	0.12
Mn_20_1077_4	2.91	16	0.18	0.22
Mn_30_1077_4	2.83	11	0.30	0.37
Mn_20_1077_3	3.19	10	0.18	0.21
Mn_20_1077_9	3.08	9	0.06	0.29
Mn_20_1077_12	2.94	7	0.05	0.50
